# Insulin Therapy with Personal Insulin Pumps and Early Angiopathy in Children with Type 1 Diabetes Mellitus

**DOI:** 10.1155/2013/791283

**Published:** 2013-11-14

**Authors:** Joanna Tołwińska, Barbara Głowińska-Olszewska, Artur Bossowski

**Affiliations:** Department of Pediatrics, Endocrinology, Diabetology with Cardiology Division, Medical University of Białystok, Białystok University Children's Hospital, 17 Waszyngtona Street, 15-274 Bialystok, Poland

## Abstract

*Objective*. Assessment of the effect of a treatment method change from multiple daily insulin injection (MDI) to continuous subcutaneous insulin infusion (CSII) on the development of early angiopathy in children with T1DM with or without retinopathy. *Methods*. The study pump group involved 32 diabetic children aged 14.8, with the initial HbA_1_c level of 8.3%, previously treated by MDI. The patients were examined before pump insertion and after 3 and 6 months of CSII. We assessed HbA_1_c level, carotid artery intima-media thickness (c-IMT), and flow-mediated dilatation (FMD) of the brachial artery. The pump group was compared to a group of eight teenagers with diagnosed nonproliferative retinopathy, treated with MDI. *Results*. HbA_1_c in the entire group was found to improve in the second and in the third examination. During 6 months of CSII, FMD increased and IMT decreased. Retinopathic adolescents had significantly thicker IMT and lower FMD compared to baseline results of the pump group. Treatment intensification in the retinopathy-free children enhanced these differences. *Conclusions*. CSII is associated with lower IMT and higher FMD. Whether on the long-run CSII is superior to MDI to delay the occurrence of diabetes late complications remains to be explained.

## 1. Introduction

Insulin pump therapy in the form of continuous subcutaneous insulin infusion (CSII) was introduced in the 1970s and turned out to ensure better metabolic control of diabetes compared to traditional insulin therapy approaches. It is currently considered to be the optimal method of insulin administration, since it imitates the pancreatic activity in the best possible way, ensures precise dosage, and at the same time offers a high level of ease and comfort. Patients that experience the advantages are above all children and adolescents with type 1 diabetes mellitus (T1DM) [1–3]. Compared to the traditional methods of insulin therapy by means of multiple daily insulin injections (MDI), CSII can significantly decrease glycated hemoglobin (HbA_1_C), reduce 24-hour glucose variability, decrease incidence of severe hypoglycemia, and eliminate dawn phenomenon. Moreover, the use of CSII is associated with improved quality of life and precise insulin administration with respect to physical effort and diet [1, 4]. A long-term effect of CSII on the development of late complications of diabetes in the form of microangiopathy or macroangiopathy needs to be explained [5].

T1DM increases the risk of developing micro- and macrovascular complications which in turn will have a devastating impact on quality of life and challenge health services worldwide. Overt microvascular complications (proliferative retinopathy, nephropathy, and neuropathy) remain rare in adolescent population with type 1 diabetes. Pathological changes, however, can occur soon after the diagnosis of type 1 diabetes and early indications are clinically detectable in adolescents after 2 to 5 yrs disease duration [6]. Screening protocols in adolescents, therefore, aim to identify the earliest signs of microvascular complications when patients at highest risk can be targeted for intensive therapy to delay the onset or slow the progression to overt end-organ complications [7]. Macroangiopathic lesions develop relatively early and clinically symptomatic ischemic heart disease may already affect young adults; heart infarcts are diagnosed in patients under 30 years of age. Microvascular complications develop parallel to macrovascular lesions and patients with microangiopathy show higher risk of ischemic diseases of the heart [8]. 

The pathogenesis of diabetic retinopathy and atherosclerosis is insufficiently understood. It is accepted that hyperglycaemia is a major causative factor for the development of these complications. There is also growing evidence for the role of low-grade inflammation and endothelial dysfunction. Circulating and locally produced inflammatory biomarkers, such as cell adhesion molecules, proinflammatory cytokines, and c-reactive protein have been shown to be of key importance for both—micro and macroangiopathy [9–11]. 

Impaired endothelial function is now considered an early sign of atherosclerosis in children, which precedes the atherosclerotic plaque formation and has therefore become an important marker of cardiovascular risk, particularly in those with T1DM. It can be detected years before overt coronary artery disease occurs [12]. Previously, we found less advanced early atherosclerotic changes in T1DM children and adolescents using insulin pumps as compared to those treated with multiple daily insulin injections. We performed ultrasound investigations of the carotid artery intima-media thickness (IMT) and flow mediated dilatation (FMD) of the brachial artery in response to reactive congestion up to passive congestion [13]. However, research has never been conducted into the effect of a change from the multiple daily injections (MDI) to CSII in the aspect of early angiopathy lesions. Therefore in this study we hypothesized, that intensification of insulin therapy in the form of CSII may have beneficial effect on the development of early micro- and macrovascular changes in young patients with T1DM. 

## 2. Objective

The study objective was, firstly, to assess the impact of treatment intensification by the use of CSII in T1DM, complication-free, children previously treated by MDI on the development of the earliest atherosclerotic lesions and, secondly, to compare the results with the group complicated by early retinopathy treated with the MDI method. 

## 3. Material and Methods

The study pump group involved 32 children (20 girls and 12 boys) with the diagnosis of type 1 diabetes, who had been treated by multiple daily insulin injections and who were qualified for CSII, in the Department of Pediatrics, Endocrinology, Diabetology with Subdivision of Cardiology, Medical University of Białystok, and in the Outpatient Diabetology Department. At baseline, the patients were at the age of 9–18 years—mean 14.8 ± 2.59, mean disease duration being 3.8 ± 3.2 years. The inclusion criterion was insulin demand of at least 0.5 IU/kg body weight/day. Patients were excluded from the study if they were obese or had hypertension, lipid disorders, and other diseases that could affect the outcome, for example, systemic connective tissue disorders, autoimmune thyroid diseases, or celiac disease. None of the patients admitted to cigarette smoking or had significant, clinically apparent diabetic complications. Patients with albuminuria were not included into this group. Retinopathy group consisted of 8 (5 boys, 3 girls) patients, aged 17.7 ± 1.4, with HbA_1_c level 9.7 ± 2.1% and disease duration of 11 ± 2.7 years. All these patients had recognized nonproliferative retinopathy, by the experienced ophtalmologist with full examination, and were treated with MDI method. The data of retinopathy group were taken from our previous studies [14].  Table 1 presents the characteristics of study pump group profile and the adolescents with diabetic retinopathy.

 The ultrasound investigations used a Hewlett Packard SONOS 4500 imaging system, with an ultrasonic transducer emitting signals at a wavelength of 7.5 MHz, according to the previously presented methodological assumptions [15, 16]. The thickness of the common carotid artery was examined as the intima-media thickness (IMT) complex. The IMT was assessed at baseline (before personal insulin pump insertion—insulin pump group 1) and after 6 months of CSII (insulin pump group 3). Nitric oxide-dependent flow-mediated dilatation (FMD) of the brachial artery was assessed after reactive congestion up to passive congestion. FMD was assessed at baseline (insulin pump group 1), after 3 (insulin pump group 2) and after 6 months of CSII application (insulin pump group 3). In retinopathy group patients, IMT and FMD were assessed by the same researcher (JT), using the same method, and compared to the values obtained in the pump study group at baseline, 3 and 6 months after the change of treatment.

The level of HbA_1_c was determined by high-pressure liquid chromatography (HPLC) and measured at baseline, after 3 and after 6 months. Results of ultrasound investigations were analyzed in the entire study group. Then, depending on the initial value of HbA_1_c the group was subdivided into well controlled, with HbA_1_c < 7.5%, mean 7.28 ± 1.36% and poorly controlled, with initial HbA_1_c > 7.5%, mean 8.88 ± 1.65% (*P* = 0.009), and both subgroups were compared as regarding IMT and FMD.

## 4. Statistical Analysis

The statistical analysis was performed using the Statistica 9.0 software (Cracow, StatSoft). The Student *t*-test was applied to determine the differences between the study groups for variables with normal distributions. The Student *t*-test for linked variables was used to compare the variables within the respective groups at baseline and after 3 and 6 months. Since in the current study the variables satisfied the conditions of normal distribution, no other tests were applied. Results were presented as the mean and standard deviation. Differences at *P* < 0.05 were considered statistically significant. 

The study was approved by the Bioethics Committee, Medical University of Białystok. Parents and children were informed about the purpose and nature of the study. The parents gave a written consent, whereas the children expressed a spoken consent before examination. 

## 5. Results

**Figure 1:**
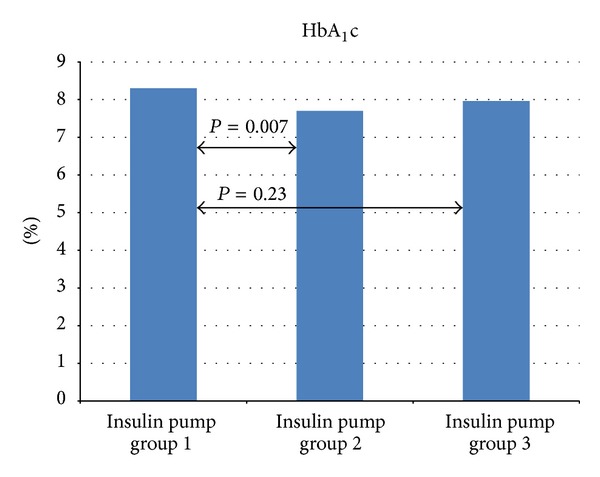
Change in HbA_1_c in the insulin pump group during 6 months of using new treatment method (1—before CSII, 2—after 3 months of CSII, and 3—after 6 months of CSII).

**Figure 2:**
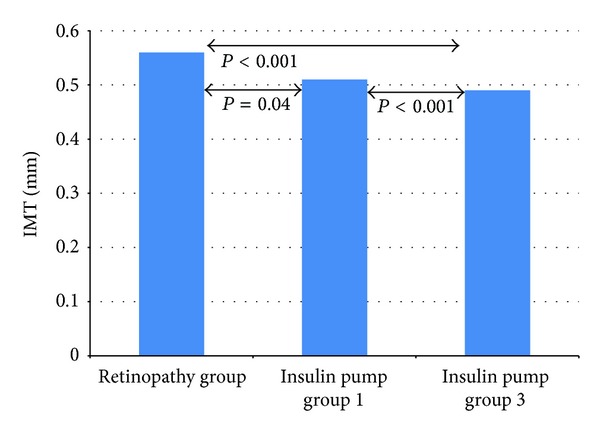
IMT values in the study groups: retinopathy group compared to the pump group before implementation of CSII (insulin pump group 1) and after 6 months (insulin pump group 3).

**Figure 3:**
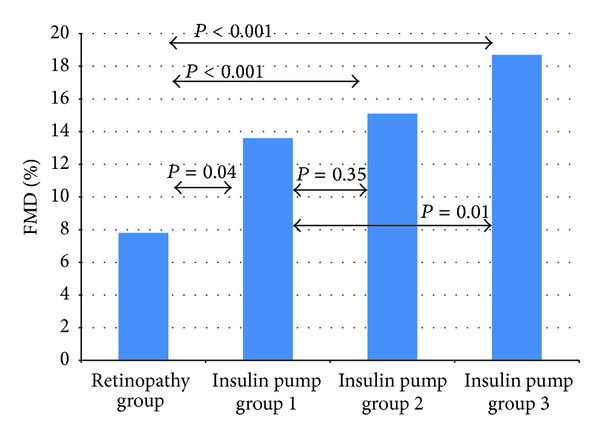
FMD values in the study groups: retinopathy group compared to the pump group before implementation of CSII (insulin pump group 1), after 3 months (insulin pump group 2) and after 6 months (insulin pump group 3).

In the study pump group, the implementation of CSII was associated with improved metabolic control. The level of HbA_1_c decreased after 3 months from 8.3 ± 1.72% to 7.7 ± 1.48% (*P* = 0.007) and after 6 months was 7.96 ± 1.46% on average (*P* = 0.23) (Figure 1). The level of IMT after the 6-month use of personal insulin pumps decreased from 0.51 ± 0.05 mm to 0.49 ± 0.04 mm (*P* < 0.001) (Figure 2). An increase was noted in FMD in the brachial artery from 13.59 ± 7.57% to 15.10 ± 7.57% after 3 months (*P* = 0.35) and to 18.75 ± 9.12% after 6 months (*P* = 0.01) (Figure 3).

**Figure 4:**
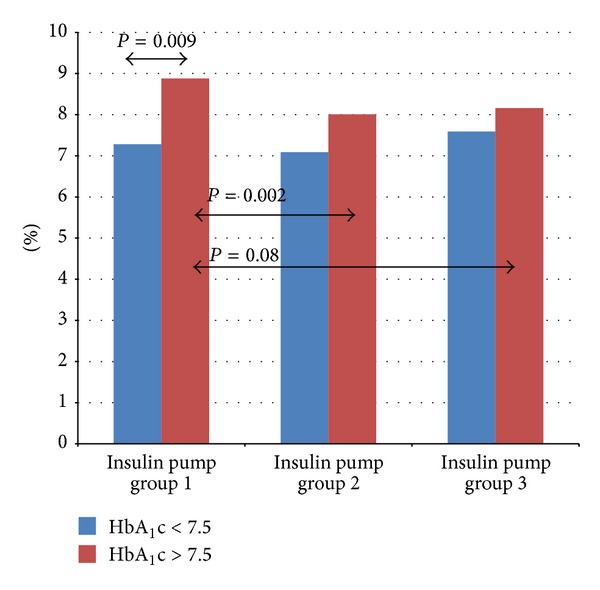
A change in HbA_1_c in the groups depending on the initial value <7.5% and >7.5% (1—before CSII, 2—after 3 months of CSII, and 3—after 6 months of CSII).

**Figure 5:**
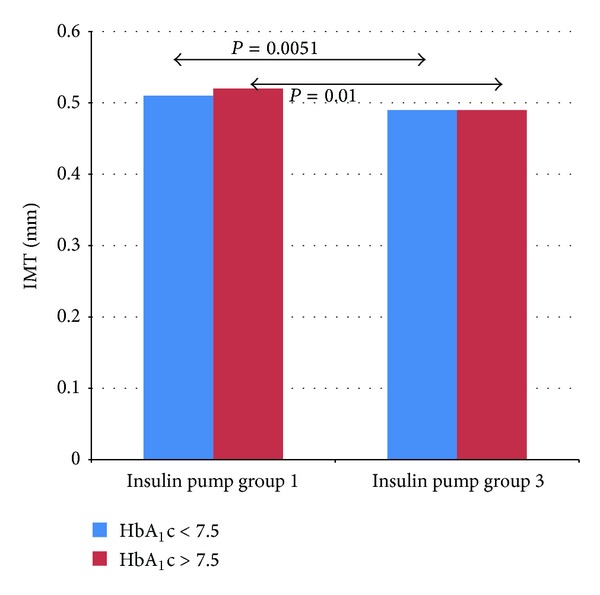
A change in IMT in the groups depending on the initial value of HbA_1_c < 7.5% and >7.5% (1—before CSII and 3—after 6 months of CSII).

**Figure 6:**
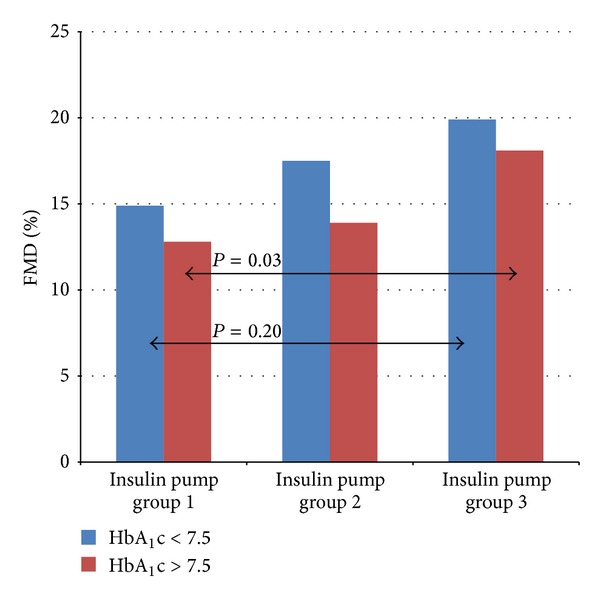
A change in FMD in the groups depending on the initial value of HbA_1_c < 7.5% and >7.5% (1—before CSII, 2—after 3 months of CSII, and 3—after 6 months of CSII).

In the well-controlled subgroup of patients (HbA_1_c < 7.5%) after 3 months the level of glycated hemoglobin decreased to 7.09 ± 1.3% (*P* = 0.64), and after 6 months its value reached 7.59 ± 1.54% (*P* = 0.48). IMT in this group was reduced significantly from 0.51 ± 0.06 mm at baseline to 0.49 ± 0.03 mm after 6 months (*P* = 0.0051), whereas mean FMD rose from 14.97 ± 5.87% to 17.51 ± 11.28% after 3 months (*P* = 0.449) and to 19.94 ± 11.58% after 6 months (*P* = 0.20). In subgroup of patients with poor metabolic control (initial HbA_1_c above 7.5%), the level of glycated hemoglobin fell from 8.89 ± 1.65 to 8.01 ± 1.49% after 3 months (*P* = 0.002) and reached the level of 8.16 ± 1.42% after 6 months (*P* = 0.08). The mean value of IMT in this group diminished from the initial 0.52 ± 0.05 mm to 0.49 ± 0.04 mm after 6 months (*P* = 0.01) and FMD increased from 12.86 ± 8.27% at baseline to 13.9 ± 4.9% after 3 months (*P* = 0.84) and to 18.18 ± 7.96% (*P* = 0.03) after half a year.  Figure 4 presents changes in HbA_1_c in the groups depending on the initial level below and above 7.5%. Figures 5 and 6 show the changes in IMT and FMD according to this criterion. Retinopathic adolescents had significantly worse values of IMT (mean 0.56 ± 0.06 mm) and FMD (7.8 ± 4.1%), the differences being even greater after 6 months of treatment intensification in the pump group (retinopathy-free ones) (Figures 2 and 3).

## 6. Discussion

T1DM is an acknowledged risk factor of atherosclerosis, and arterial lesions, including the coronary ones, are already found in children. Their severity correlates with the level of glycated hemoglobin [17, 18]. Up to now, many researchers have investigated the correlations of disease duration, age at onset, metabolic control, and insulin doses with markers of early atherosclerosis (IMT, FMD) in ultrasound imaging [19–22]. Pozza et al. in a two-year observation of diabetic children found greater progress of IMT in patients with higher levels of HbA_1_c and systolic pressure [23]. In children with diabetes, the levels of IMT and FMD are affected significantly by the presence of complications and additional risk factors, such as obesity, hypertension, or lipid metabolic disorders [24]. According to Margeirsdottir et al., in diabetic children additional risk factors of cardiovascular diseases co-occur at 84% (one factor) and 15% (three factors) [25]. Our patients in the present study, however, had no burden of any other risk factors of atherosclerosis that could affect the study outcome. 

In the 20-year study of 1.604 adolescents with diabetes type 1, the prevalence of retinopathy has continued to decrease in parallel with the decline in HbA_1_c and intensification management. Although the discussed study was not an interventional one, authors confirmed a contemporary association among intensive management, improved glycemic control, and lower risk of retinopathy, more than 20 years after the DCCT was performed [26]. Authors findings provide some reassurance for lower glycaemic targets and increased use of MDI and CSII in children and adolescents with diabetes type 1 [27]. Retinopathy was found in approximately half of adolescents with type 1 diabetes after a median duration of 9 years in the early 1990s, compared with only 12% in recent years. It is of interest that in subgroups analysis of patients treated only with intensive management, there was some evidence for reduced risk for retinopathy in those treated with CSII compared to MDI. Given there was no difference in HbA_1_c between groups author of the mentioned study hypothetized that reduced glycaemic variability may have contributed to this difference. There are no studies demonstrating a specific benefit of CSII over MDI on complications in adolescents. However, there are now more patients than ever reaching the recommended HbA_1_c below 7.5% [27]. 

Our nowadays patients population turned to change similarly to the abovementioned ones we do not observe any form of retinopathy in our young patients. As the main difference between past and actual patients is the insulin regimen pattern, with broad use of CSII, we believe that wide range application of personal insulin pumps in actual cohort might influence the clinical picture of complication-free ones. To compare the data of early angiopathy results, such as IMT and FMD in young patients with retinopathy, we had to make use of the results from previous studies [14]. 

We presented earlier a preliminary report of our results concerning the impact of treatment intensification on the development of the earliest atherosclerotic lesions in type 1 diabetes in children [13]. The current study differs from the previous one in that it involved one group of patients who underwent a change in the mode of insulin administration, from MDI to CSII. The 6-month observation on a larger group than previously described showed beneficial effects of CSII. We found a significant correlation of IMT and FMD with metabolic control. Interestingly, the children who were initially poorly controlled, during CSII therapy, were close to reach the target HbA_1_c and FMD levels observed in well-controlled patients, which can be explained by, for example, better motivation to follow the therapeutic regimen. Lack of similar correlation with regard to IMT can result from its lower liability.

After 3 and 6 months, there was a drop in HbA_1_c levels, which may be associated with treatment intensification and better adjustment of insulin doses to physiological needs of the body. Slight exacerbation of metabolic control after 6 months as compared to 3 months of treatment is likely to result from poorer self-control of patients who got used to the new method. This correlation was more distinct in the group of children with better metabolic control—the mean HbA_1_c value exceeded slightly the initial value. This may virtually indicate that children should be reeducated after the first few months of insulin pump application.

In our study, retinopathic adolescents had significantly worse values of IMT and FMD, the differences being even greater after 6 months of treatment intensification in the study group (retinopathy-free ones). The results of this study display profits from CSII for adolescents suffering from type 1 diabetes with this complication nowadays. In the study by Downie et al. both retinopathy and microalbuminuria were associated also with an improvement in socioeconomic status over time. It is possible that there has been a bias towards higher socioeconomic status individuals who attend a complications assessment in recent years, in parallel with improved knowledge [27]. 

In recent years, a hypothesis has been formulated that even at similar levels of glycated hemoglobin, the risk of diabetic complications is higher in patients with greater glycemic variability, increased oxidative stress, and substantial glucose fluctuations. The increased oxidative stress can be strictly linked to impaired endothelium-dependent vasorelaxation [28–30]. In T1DM patients, including children, oxidative stress is a well-known phenomenon [31]. Bruttomesso et al. have proven that young adults with type 1 diabetes are characterized by better metabolic control, lower glucose variability, fewer hyperglycaemic episodes, less daily insulin dose, and lower free fatty acids when treated with CSII as compared to those treated with MDI. CSII has been found to reduce glycemic variability as compared to MDI also in other studies [32–34]. In the study by Schreiver et al. the authors were not able to prove that together with observed reduced glycaemic variability during CSII compared with MDI, markers of oxidative stress also decrease. Authors underlined, however, methodological problems with estimating oxidative stress markers and a possible missing link between glycaemic variability and oxidative stress in T1DM patients [35]. A new trace in this subject may bring the conclusions from the recent study, where recurrent hypoglycemia episodes were recognized as a risk factor for future cardiovascular disease [36]. Reduced glycemic variability with lower hypos on pumps therapy can be of great importance. There is also growing evidence that CSII decreases cardiovascular risk factors in poorly controlled type 2 diabetes [37], suggesting that synchronization of sufficient insulin peaks with meal ingestion and continuous pulsatile infusion of basal insulin corrects metabolic derangements. As there are no such data considering T1DM young patients, a further search for correlations between advancement of micro- and/or macroangiopathy, intensification of treatment with use of CSII and glycemic variability in children and adolescents may appear a challenge.

*Limitations of the Study.* There are certain limitations of our study implicating a careful interpretation of the conclusions. The sample size was relatively small limiting power for analysis. The group with retinopathy was older and with longer diabetes duration, which might influence the statistical significance in the differences observed in our results. Despite these limitations we believe our data may still contribute to understand some possible mechanisms of the impact of CSII on the early angiopathy risk in T1DM young patients.

## 7. Conclusions

The application of CSII through treatment intensification improves ultrasound imaging parameters (IMT, FMD) in the assessment of early atherosclerotic lesions in children with type 1 diabetes. Baseline higher glycated hemoglobin is associated with the possibility of achieving better effects of treatment intensification. In patients with initially proper metabolic control, the use of personal insulin pumps has a beneficial effect on the earliest stages of atherosclerosis in the assessment of IMT and FMD, even despite lack of significant improvement in HbA_1_c. Whether on the long-run CSII is superior to MDI to delay the occurrence of diabetes late complications remains to be explained in future trials.

## Figures and Tables

**Table 1:** General characteristics of the insulin pump group and retinopathy group.

	Insulin pump group *n* = 32	Retinopathy group *n* = 8	*P* value
Age (years)	14.9 ± 2.5	17.7 ± 1.4	0.057
Gender: boys/girls	12/20	5/3	0.600
Height (cm)	163 ± 10	160 ± 10	0.870
Body mass (kg)	58 ± 10	66.4 ± 12.7	0.080
BMI (kg/m^2^)	21.6 ± 2.8	25.2 ± 3.2	0.002
SDS-BMI	0.6 ± 0.9	1.3 ± 1.2	0.072
Diabetes duration (years)	3.7 ± 3.2	11 ± 2.7	<0.001
HbA_1_c (%) (before pump therapy)	8.3 ± 1.7	9.7 ± 2.1	0.057
HbA_1_c (%) (6 months after pump therapy)	7.9 ± 1.4	9.7 ± 2.1	0.008
Systolic blood pressure (mmHg)	115 ± 7	133 ± 18	<0.001
Diastolic blood pressure (mmHg)	69 ± 6	76 ± 10	0.020
Total cholesterol (mg/dL)	163 ± 27	179 ± 33	0.159
LDL-cholesterol (mg/dL)	82 ± 21	105 ± 22	0.011
HDL-cholesterol (mg/dL)	62 ± 12	47 ± 7	0.003
Triglicerides (mg/dL)	72 ± 21	129 ± 64	<0.001
